# Energetics of Transport through the Nuclear Pore Complex

**DOI:** 10.1371/journal.pone.0148876

**Published:** 2016-02-19

**Authors:** Ali Ghavami, Erik van der Giessen, Patrick R. Onck

**Affiliations:** Zernike Institute for Advanced Materials, University of Groningen, Groningen, the Netherlands; Russian Academy of Sciences, Institute for Biological Instrumentation, RUSSIAN FEDERATION

## Abstract

Molecular transport across the nuclear envelope in eukaryotic cells is solely controlled by the nuclear pore complex (NPC). The NPC provides two types of nucleocytoplasmic transport: passive diffusion of small molecules and active chaperon-mediated translocation of large molecules. It has been shown that the interaction between intrinsically disordered proteins that line the central channel of the NPC and the transporting cargoes is the determining factor, but the exact mechanism of transport is yet unknown. Here, we use coarse-grained molecular dynamics simulations to quantify the energy barrier that has to be overcome for molecules to pass through the NPC. We focus on two aspects of transport. First, the passive transport of model cargo molecules with different sizes is studied and the size selectivity feature of the NPC is investigated. Our results show that the transport probability of cargoes is significantly reduced when they are larger than ∼5 nm in diameter. Secondly, we show that incorporating hydrophobic binding spots on the surface of the cargo effectively decreases the energy barrier of the pore. Finally, a simple transport model is proposed which characterizes the energy barrier of the NPC as a function of diameter and hydrophobicity of the transporting particles.

## Introduction

Molecular transport between the cytoplasm and the nucleoplasm is gated by highly selective nuclear pore complexes (NPCs). The NPC is embodied in the nuclear envelope membranes and provides bidirectional pathways for passive transport of small molecules and active (facilitated) transport of larger molecules [1–4]. Small molecules and ions are passively transported through the pore by free diffusion, but large molecules are barred to enter the pore. The transport of large macromolecules requires binding to soluble transport factors, generally known as karyopherins or Kaps. During active import or export the Kap binds to cargo with a nuclear import or export signal, upon which the Kap-cargo complex is translocated through the NPC.

The NPC of yeast, as a model system, is composed of approximately 30 different proteins called nucleoporins (Nups) which are arranged with an eight fold-symmetrical pattern inside the pore [5, 6]. The Nups that line the central channel of the pore have been found to be intrinsically disordered and contain many phenylalanine-glycine (FG) repeats [7]. These FG-Nups are essential for the viability of yeast and presumably all eukaryotes but their function in regulating the active and passive transport is not yet understood [8]. Several models have been proposed to explain the role of FG-Nups and Kaps during transport but no consensus has been reached so far on a prevailing model [3, 9–14].

The permeability barrier of the NPC has been characterized by studying the passive transport of a wide range of inert molecules of different size. Single molecule studies have revealed that cargoes up to 29 kDa can smoothly diffuse through the pore, while transport of cargoes larger than 61 kDa is prohibited [15]. Early experiments have estimated a diameter of 4.5–5.4 nm for the diffusion channel of the NPC [16, 17], which was refined to a diameter of ≈ 5.3 nm in more recent experiments [18]. In addition to the size, the shape of the transporting species has been shown to be an important factor in passive transport. It has been observed that elongated non-spherical cargoes diffuse faster than spherical ones with the same mass [18]. Passive and active transport pathways have been widely discussed in the literature [12, 15, 18, 19]. Several studies have suggested that passive and active transport take place through different spatially-separated pathways across the pore [15, 19, 20]. Recently, it has been demonstrated that passive transport mostly occurs through the central region of the pore [15, 21].

Mutational analyses of Kaps suggest that the interaction between Kaps and FG-repeats is necessary for active transport [22, 23]. It has been shown that Kaps have a greater surface hydrophobicity compared to other cytoplasmic proteins [24]. In addition, structural analyses have demonstrated that, during interaction, hydrophobic side chains of the FG-Nups closely interact with hydrophobic pockets on the surface of the Kap [22, 25]. Naim and co-workers [26] have shown that a cargo that is normally blocked, can be triggered to enter by modification of its surface with hydrophobic amino acids. This suggests that a certain amount of surface hydrophobicity is necessary for cargo to actively translocate through the pore. Molecular dynamics simulations and experiments have revealed several binding spots on the surface of Kaps [27–30]. Recently, the effect of electrostatic interactions on active and passive transport has been studied through a high resolution fluorescence microscopy technique [31]. The results of this experiment suggest that electrostatic interactions are less important than hydrophobic interactions in nuclear transport through the NPC.

In addition to experimental studies, several theoretical and computational studies have been conducted to elucidate the mechanism of nuclear transport. These studies include investigations of single FG-Nups [12, 32], their collective behavior in brush-like structures [33, 34] and in the transport channel of the NPC [35–37] as well as modeling transport through the nuclear pore [38–41]. Regarding nuclear transport, Mincer et al. [38] have used a super-resolution approach which treats FG-Nups as flexible filaments with various binding spots to serve as FG-repeats. Even though many simplifying assumptions are made, the model can qualitatively predict several aspects of transport. Coarse-grained Brownian dynamics simulations have shown that the Kap-cargo complex interacts with a layer of FG-Nups formed close to the channel wall [39]. However, the specific amino acid sequence of the FG-Nups was not accounted for in these simulations. The density distribution of FG-Nups and colloidal particles inside a cylindrical axi-symmetric structure, mimicking the NPC, have been studied through a classical density functional theory approach [41]. The model provides valuable insight into several aspects of nuclear transport including the crowding effect of the cargoes inside the pore. The energetics of translocation of a model cargo through the pore has been investigated by Tagliazucchi and coworkers [40] using a theoretical model that accounts for different FG-Nups by distinguishing six families of amino acids. Their results suggest that the transporting cargo experiences an energy barrier at the center of the pore which is lowered through hydrophobic and electrostatic interactions. However, the size selectivity of the NPC during passive transport and the effect of binding spot distribution on active transport have not been investigated.

We have previously investigated the distribution of disordered FG-Nups in the transfer conduit of the NPC by means of coarse-grained implicit solvent molecular dynamics sumulations [37]. The developed one-bead-per-amino-acid model distinguishes between all 20 amino acids of the FG-Nups and takes into account hydrophobic and electrostatic interactions between the amino acids, the backbone stiffness of the Nups as well as the screening effect of free ions and polarity of the solvent through a modified Coulomb equation. The model has been calibrated against experimental Stokes radii of a wide range of FG-Nup segments [12]. Since the model is parametrized against the equilibrium properties of FG-Nups, it cannot be directly used to study kinetic aspects of nuclear transport. The goal of the present work is to use this model to study the energetics of passive and active transport through the disordered domain of the nuclear pore complex. We examine the size selectivity of the NPC by calculating the energy barrier for passively transporting cargoes. In addition, we elucidate the active transport mechanism by studying how the number of binding spots and their spacing on the surface of model Kaps affect the energy barrier.

## Methods

Molecular dynamics simulations are performed using a one-bead-per-amino acid coarse-grained (CG) model [37]. The distance between neighboring beads is fixed at 0.38 nm using a harmonic potential and an average mass of 120 Da is assigned to each CG bead. The backbone stiffness of the FG-Nups is controlled through bending and torsion potentials extracted from the Ramachandran data of the coiled regions of protein structures [42]. The Gromacs molecular dynamics software [43] is used to perform Langevin dynamics simulations. The temperature is set to 300 K and the cut-off distance for Van der Waals and Coulombic interactions are set to 2.5 nm and 5.0 nm, respectively. A time-step of 0.02 ps is chosen and the Langevin friction coefficient is set to 50 ps^−1^ which is similar to the collision frequency of water molecules [44]. For details of the model the reader is referred to Ghavami et al. [37].

**Fig 1:**
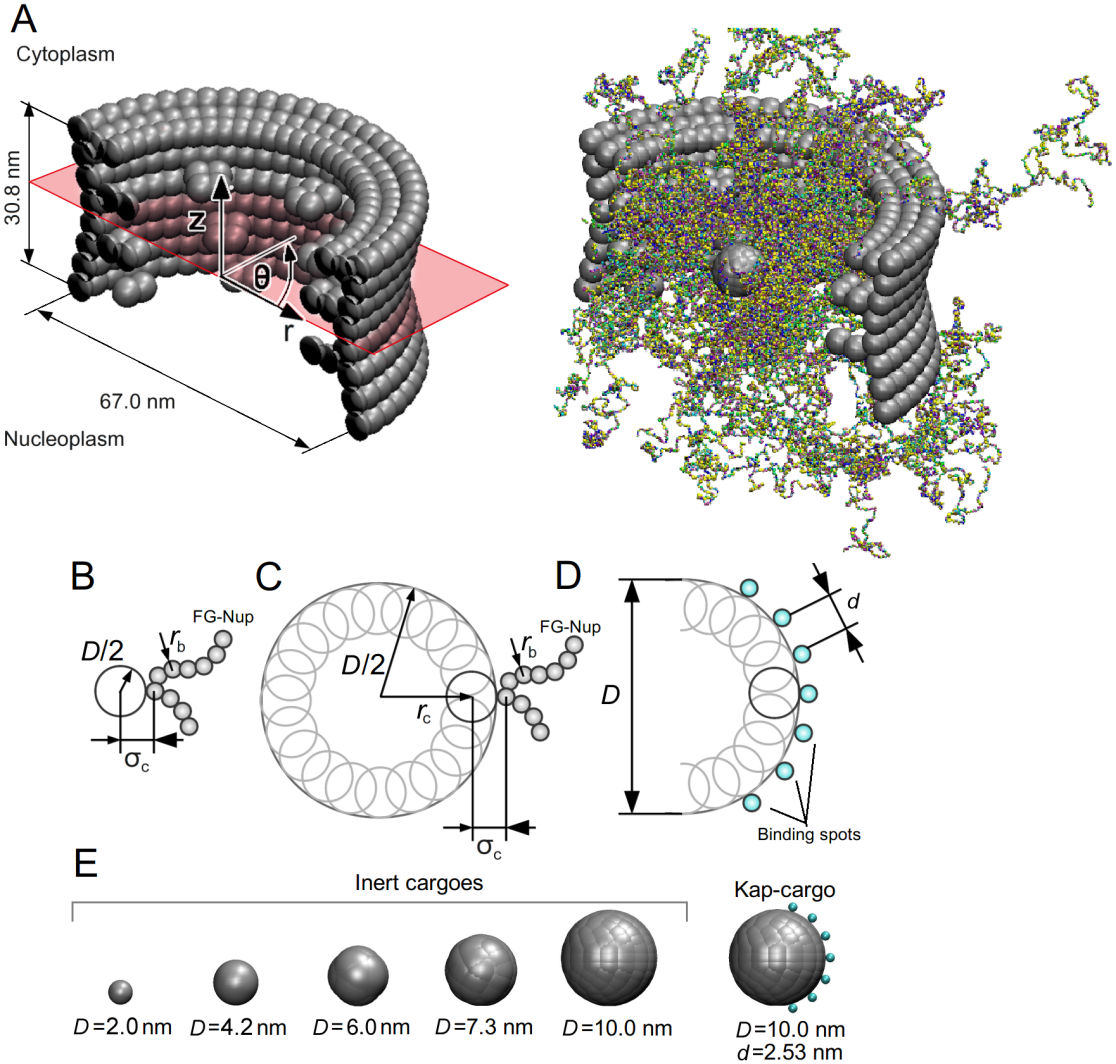
The geometrical model of the NPC and cargoes. (A) (left) The core scaffold of the NPC, reconstructed based on the structural model proposed in [45], (right) a snapshot taken from an umbrella sampling simulation for a cargo with *D* = 10 nm. (B) Geometrical representation of a model cargo smaller than 5.0 nm in diameter and (C), composite cargoes larger than 5.0 nm. (D) Geometrical representation of a model Kap-cargo complex with 7 binding spots. (E) The inert cargoes with different diameters and a Kap-cargo complex as used in the simulations.

A simplified geometrical model of the NPC is built based on the geometry of the core scaffold of the yeast NPC and the FG-Nups are anchored at the predicted positions inside the pore [45, 46]. The scaffold is modeled using hard-sphere beads with a diameter of 5.0 nm which are assumed to have no interaction with the FG-nups (see Fig 1A).

Passively transporting cargoes are modeled as rigid spheres with diameter *D*. Inert cargoes smaller than 5.0 nm in diameter are modeled as a single neutral bead with a purely repulsive potential at distances smaller than *σ*_*c*_ = *D*/2+*r*_*b*_, where *r*_*b*_ = 0.3 nm is the radius of the CG-beads for the FG-Nups (see Fig 1B). Cargoes larger than 5.0 nm in diameter are constructed with a number of neutral beads each having a repulsion distance of *σ*_*c*_ = 2.5 nm as shown in Fig 1C. These overlaping beads are centered at a distance of *r*_*c*_ = *D*/2 − (*σ*_*c*_ − *r*_*b*_) from the center of the composite cargo. A schematic representation of the inert cargoes used in this work is presented in Fig 1E.

Kaps are elongated boat-like proteins with FG-Nup binding spots on their convex surface [3, 13], while their concave surface is used to attach to the cargo. In view of this, the Kap-cargo complex is modeled as a rigid sphere of *D* = 10 nm with several binding spots on its surface. Since binding spots and FG-repeats are reported to have similar affinities with FG-Nups [47], the binding spots are represented by hydrophobic beads that are similar to Phenylalanin (F) amino acids (with a maximum interaction energy of −5.2 kJ.mol^−1^) and are distributed on the surface of the sphere along a stripe at a spacing *d* (see Fig 1D and 1E) [27].

We use umbrella sampling to calculate the potential of mean force (PMF) of the transporting cargoes [48]. The PMF is the effective potential that a particle experiences at a certain position due to the presence of all other particles, averaged over all conformations of the system. In the umbrella sampling method, the reaction coordinate is subdivided into several overlapping windows. The system is then simulated in the presence of a bias potential to enhance the sampling in each window. Ultimately, the information from the separate simulations are unbiased and recombined to obtain the PMF along the reaction coordinate (see the S1 Text).

## Results and Discussions

### Size selectivity of the NPC

**Fig 2:**
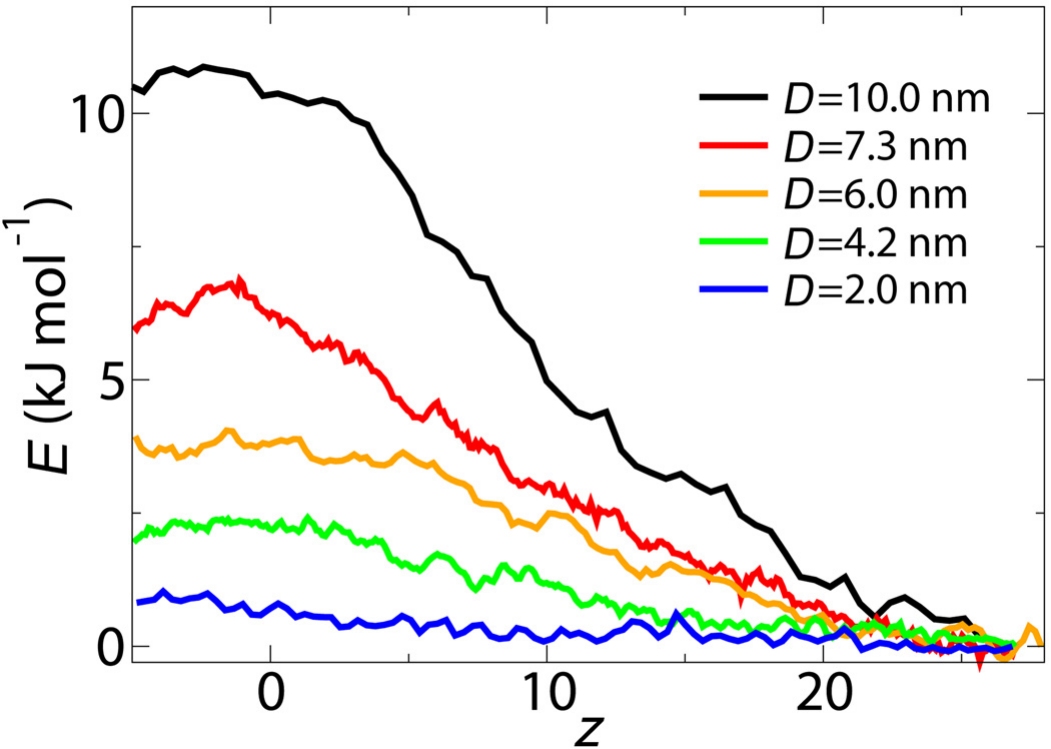
Potential of mean force curves along the central axis of the NPC (*r* = 0) for cargoes with *D* = 10, 7.3, 6.0, 4.2 and 2.0 nm.

First, the PMF curve along the central axis of the NPC is calculated for inert cargoes of different size as shown in Fig 2. In all cases the PMF curve increases as the cargo relocates from its starting position inside the cytoplasm (*z* = 27 nm) to the center of the NPC and a peak value is observed near the central plane of the NPC. This continuous increase in PMF already starts inside the cytoplasm (i.e., *z* > 15.4 nm, see Fig 1), which indicates that some of the FG-Nups extend to the cytoplasmic environment and repel large cargo molecules by their entropic motion. This is consistent with our previous study showing that the FG-Nups located at the peripheries of the NPC have more conformational freedom and are spread over a larger volume compared to the FG-Nups located near the symmetry plane of the NPC [37].

**Fig 3:**
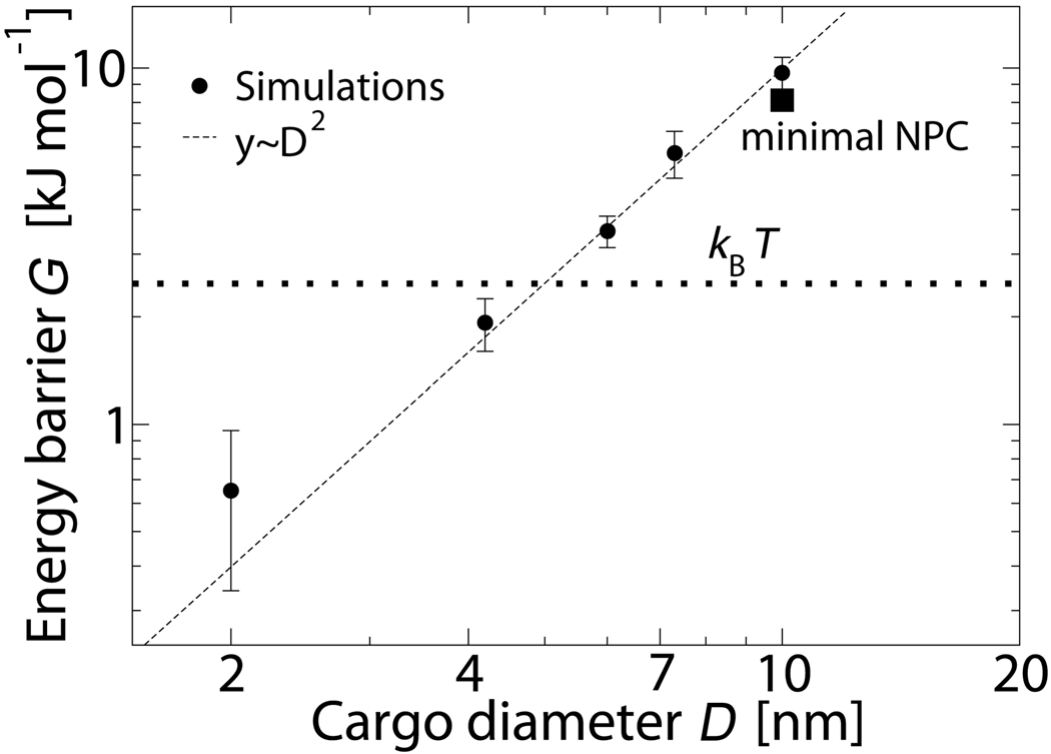
The energy barrier *G* versus diameter *D* of the cargoes. The dashed line is a quadratic fit to the data and the error bars indicate the standard deviation of the data for the interval -5.0 nm < *z* < 5.0 nm and 20 nm < *z* <27 nm.

In order to study the size selectivity of the NPC, the free energy barrier corresponding to different cargo diameters is calculated and plotted in Fig 3. The energy barrier is defined as the difference between the mean PMF at -5.0 nm < *z* <5.0 nm and 20 nm < *z* <27 nm and is an indication of the work required to translocate the cargo from the cytoplasm to the core of the NPC. The results show that the energy barrier of the NPC for passively transporting cargoes decreases as the diameter of the cargo decreases. The size selectivity threshold of the pore is calculated by comparing the energy barrier of the pore with the thermal energy *k*_*B*_*T*. If the energy barrier experienced by the cargo is larger than *k*_*B*_*T*, the probability that a cargo passes through the pore decreases. Thus, by defining *k*_*B*_*T* as a soft limit for transport, we find that cargoes larger than *D* = 5.0 nm have a small probability to pass through. This is within the experimental estimates in the range 4.5–5.4 nm for the size selectivity of the NPC [16–18].

A comparison between the energy barrier of the wildtype and a minimal viable NPC for a cargo with *D* = 10 nm is made in Fig 3. The minimal viable NPC corresponds to an NPC that has the least amount of FG-nups according to Strawn et al. [8], yet is viable. In the minimal viable NPC Nup42, Nup159, Nup1, Nup60, Nup100, Nsp1 and Nup145 are removed (see [37]). The results indicate that the minimal viable NPC is also able to screen large non-specific cargoes from entering the pore. However, the energy barrier has decreased by 22% in the minimal NPC compared to the wildtype NPC, which is probably due to the removal of half of the mass of the FG-Nups in the minimal viable NPC.

**Fig 4:**
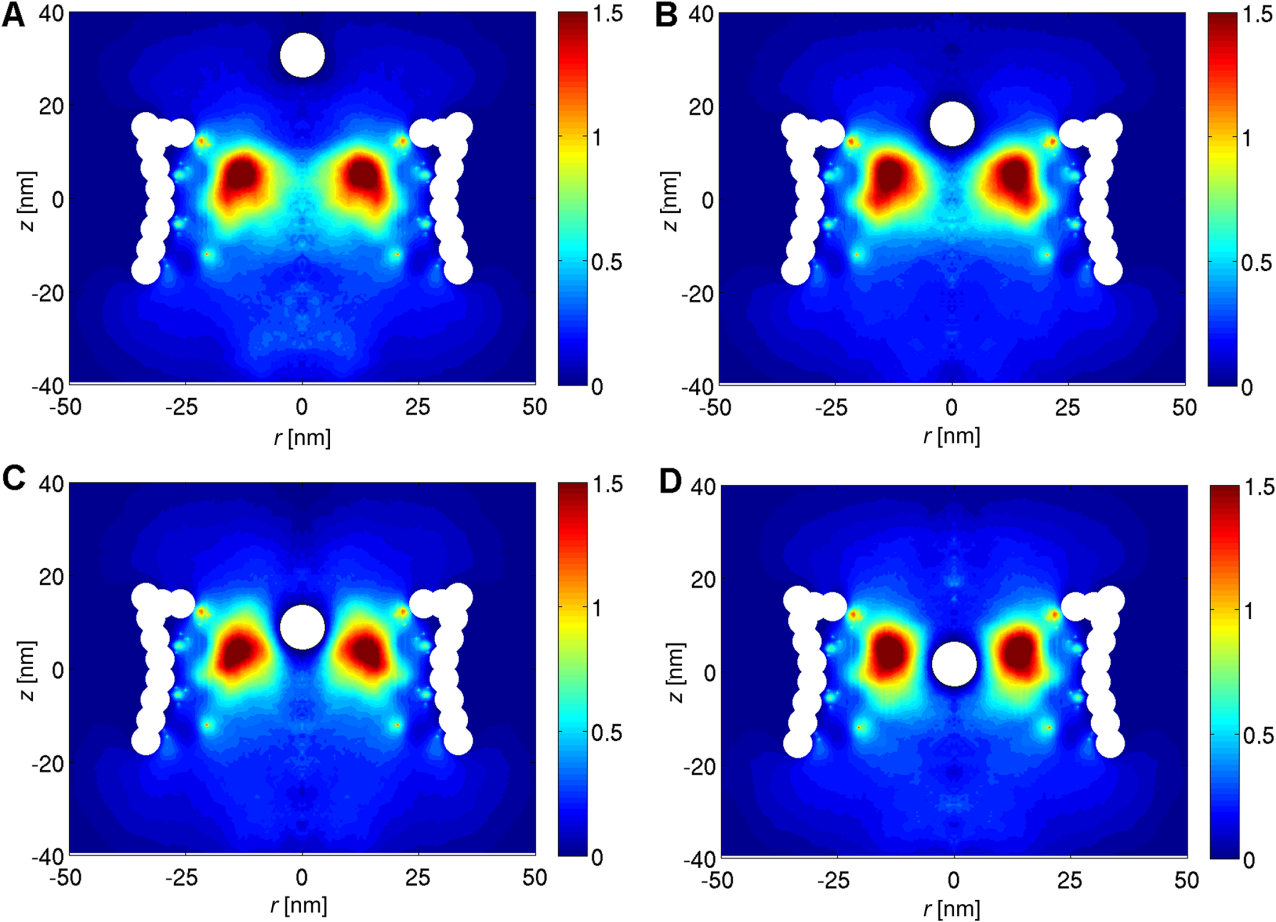
The 2D density plots of the FG-Nups taken from the umbrella simulations. The vertical distance from the center of the cargo to the central plane of the NPC is (A) 30 nm, (B) 15.6 nm, (C) 8.3 and (D) 1.1 nm, respectively.

In order to gain more understanding on the energy barrier of the NPC, the density distribution of the FG-Nups is studied in the presence of a cargo with diameter of *D* = 10 nm at different vertical distances from the central plane of the NPC (see Fig 4). The two-dimensional density plots are obtained by averaging the density distribution of the FG-Nups in the circumferential direction for the umbrella simulations [37]. The results show that once the cargo approaches the center of the NPC, it has to push the high density FG-Nup region aside in order to pass through the pore. This will result in a high energetic penalty for the cargo to pass through the central region of pore, resulting in a total energy barrier of 10.5 kJ/mole (Fig 2).

### Possible pathways

**Fig 5:**
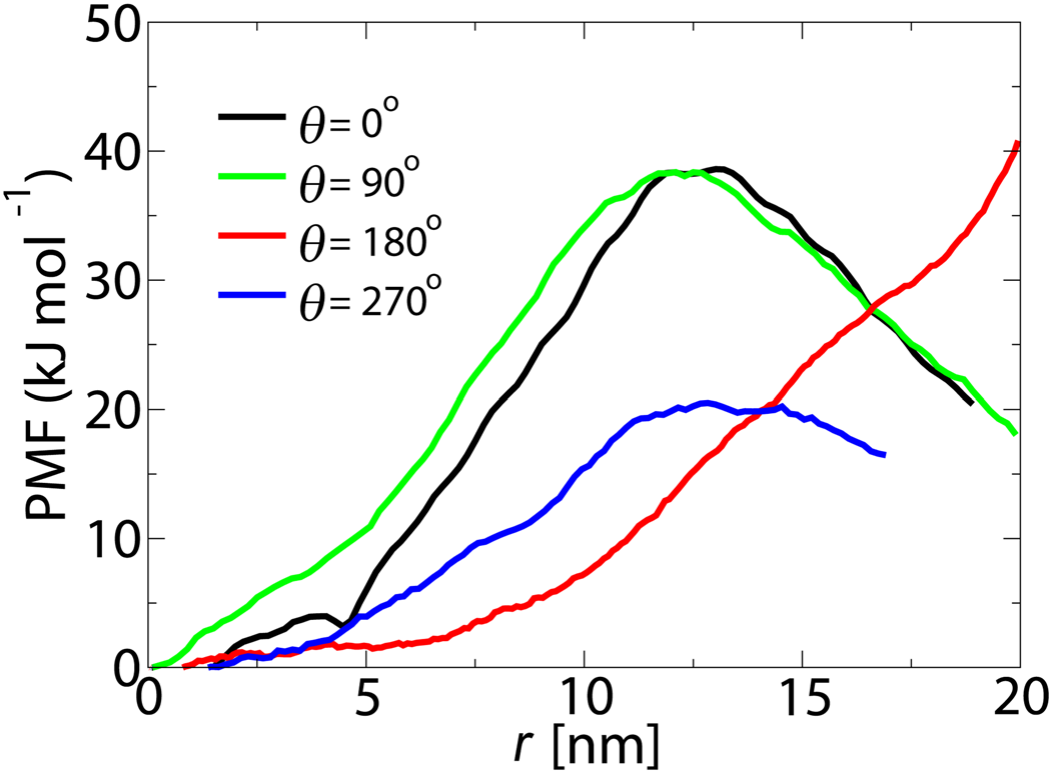
The potential of mean force along the radial direction of the NPC (at *z* = −2.5 nm) for a cargo with *D* = 10 nm.

The calculated PMF curves in Fig 2 are obtained along the central axis of the pore (*r* = 0). In order to check whether the lowest energy route for passive transport passes through the center, radial PMF curves have been calculated. This is done by comparing radial free energy profiles for a cargo with *D* = 10 nm at the maximum point of the axial PMF curve (i.e., *z* = −2.5 nm, see Fig 2). The radial energy profiles are obtained in four different directions, *θ* = 0°, 90°, 180° and 270° (see Figs 1 and 5).

The difference between the PMF curves can be rationalized by the non-uniform distribution of the FG-Nups in the pore (see [37]). For all orientations the lowest energy pathway for an inert cargo with *D* = 10 nm passes through the center of the pore. This is consistent with single molecule tracking experiments which suggest that passively transporting particles pass through the center [15].

### Lowering the barrier

**Fig 6:**
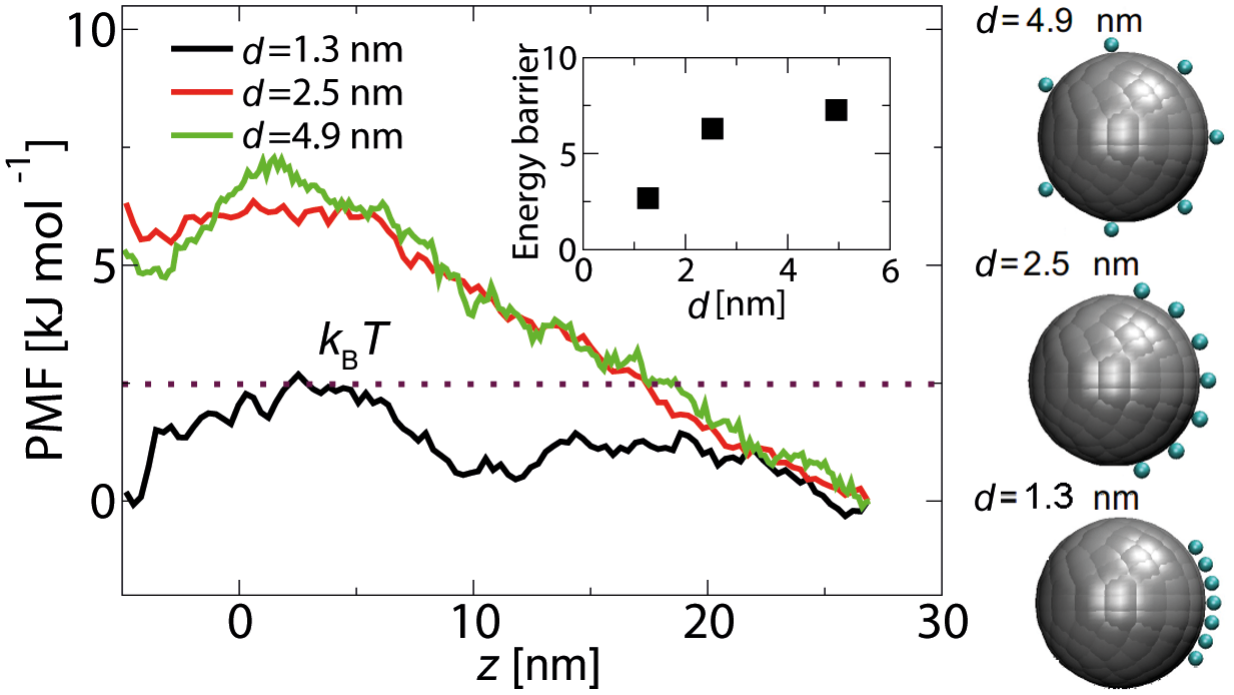
The free energy curves along the central axis of the NPC (*r* = 0), for a Kap-cargo complex of diameter *D* = 10 nm with different spacings *d* between the binding spots on its surface. The inset shows the energy barrier versus spacing *d*. The interaction energy between individual binding spots and the FG-repeats is −5.2 kJ.mol^−1^.

The energetics of active transport is investigated by calculating PMF curves for Kap-cargo complexes through the central axis of the NPC (*r* = 0 nm). The Kap-cargo complex is modeled as a sphere of diameter *D* = 10 nm with 7 binding spots on its surface, but at different spacings *d* (see Figs 1 and 6). When the binding spots are spaced at *d* = 4.94 nm, Fig 6 shows that a reduction is observed in the energy barrier of the NPC from 10.5 kJ/mol in the absence of any binding spots (cf. Fig 2) to 7.3 kJ/mol. However, this reduction is not enough for transport of the model Kap-cargo complex. When the spacing between the binding spots is further decreased to 2.5 nm, the energy barrier does not show a considerable difference from *d* = 4.9 nm. However, when the spacing is decreased to *d* = 1.3 nm, the obtained PMF curve shows a large reduction of the energy barrier to the order of *k*_*B*_*T* (see Fig 6). These trends are consistent with the experimental findings of Naim and co-workers [26], who showed that large inert cargoes were able to transport through the pore when hydrophobic amino acid side chains were attached to the surface of the cargo.

In addition, the critical spacing of 1.3 nm is in close agreement to the reported values of *d* = 1.1 ± 0.3 nm and *d* = 1.4 ± 0.3 nm for the distance between binding spots on the surface of Importin-*β* and NTF2, respectively [27].

### Effect of the number of binding spots

**Fig 7:**
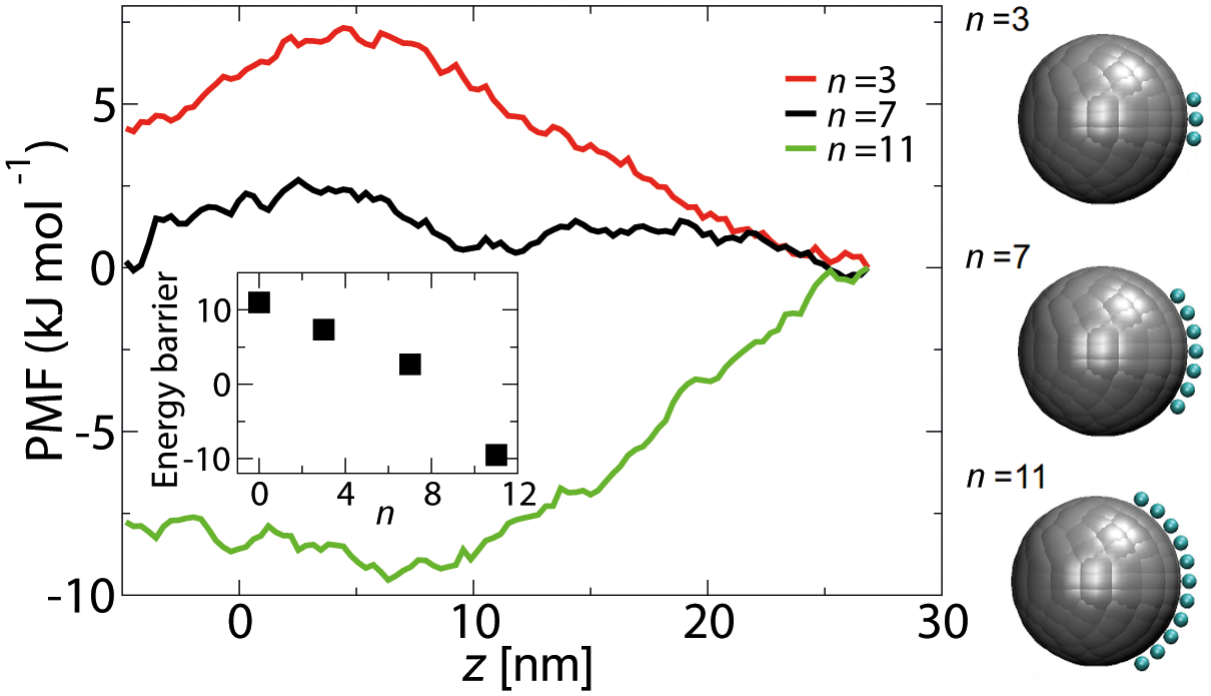
The free energy curves along the central axis of the NPC (*r* = 0), for a Kap-cargo complex of diameter *D* = 10 nm with different number of binding spots *n*. The inset shows the energy barrier versus the number of binding spots *d*. The interaction energy between individual binding spots and the FG-repeats is −5.2 kJ.mol^−1^.

In order to quantify the effect of hydrophobicity of the Kap-cargo surface on active transport, PMF curves for Kap-cargo complexes are computed for a fixed spacing (*d* = 1.3 nm), but with varying number of binding spots (i.e., *n* = 3, 7 and 11), see Fig 7. The addition of 3 binding spots decreases the energy barrier from 10.5 kJ/mol (no binding spots, see Fig 2) to 7.3 kJ/mol. For *n* = 7, the energy barrier is on the order of *k*_*B*_*T*. Interestingly, by increasing the number of binding spots to *n* = 11, the barrier completely disappears and the NPC forms a potential well for the Kap-cargo complex. In this case the complex is attracted towards the pore and tends to stay in the central region of the NPC. Clearly, the probability for transport would be strongly reduced compared to a complex with 7 binding spots.

The energy barrier versus the number of binding spots *n* is plotted in the inset of Fig 7. The results suggest that there is an optimum number of binding spots for efficient transport of a Kap-cargo complex through the pore. It must be noted, however, that our simulations are for a cargo with *D* = 10 nm and the optimum number of binding spots might be different for a cargo with different size.

### Transport model

Earlier theoretical and experimental studies propose that the free energy of insertion of particles into grafted polymer brush scales with either the volume (in high grafting densities or bad solvent conditions) or the surface area of the particle (in low grafting density or good solvent conditions) [49–52]. Since the density of the FG-nups is low in the central channel of the NPC, the free energy penalty for passage of cargoes scales with their surface area *G* ∼ *D*^2^ (see Fig 3). Upon addition of binding spots on the surface of the cargo, the free energy barrier will be reduced by an attractive energy gain [53] proportional to the contact area of the cargo and the FG-nups. Based on these considerations, a simple transport model is proposed, which describes the energy barrier of the NPC, *G*, as a function of the diameter and hydrophobicity of the Kap-cargo complex,

$$G\left(D,n\right)=f\left(D^{\prime }\right)-g\left(X\right),$$ (1)

where *D*′ = *D*/*L* is the diameter of the cargo complex normalized by the diameter of the NPC (*L* = 60 nm), and *X* = *na*/(*πD*) is a measure of the hydrophobic contact area of the cargo in terms of the number of hydrophobic binding spots, *n*, and the diameter of the binding spots *a* = 0.6 nm. The dependence on cargo size for *n* = 0 is included through a quadratic function *f*(*D*′) = *a*_1_ *D*′^2^ which is obtained by fitting the results of Fig 3. The function *g*(*X*) indicates the reduction of the free energy barrier as the hydrophobic contact area of the Kap-cargo complex is increased and is obtained by fitting the results of Fig 7 for a cargo with *D* = 10 nm to a quadratic function *g*(*X*) = *b*_2_ *X*^2^ + *b*_1_ *X* + *b*_0_ (the constants *a*_*i*_ and *b*_*i*_ can be found in the S2 Text). The accuracy of the model is verified by comparing the predicted and calculated energy barriers for a Kap-cargo complex with *D* = 7.3 nm and *n* = 5 and 8 hydrophobic binding spots (see S3 Fig), showing good agreement.

**Fig 8:**
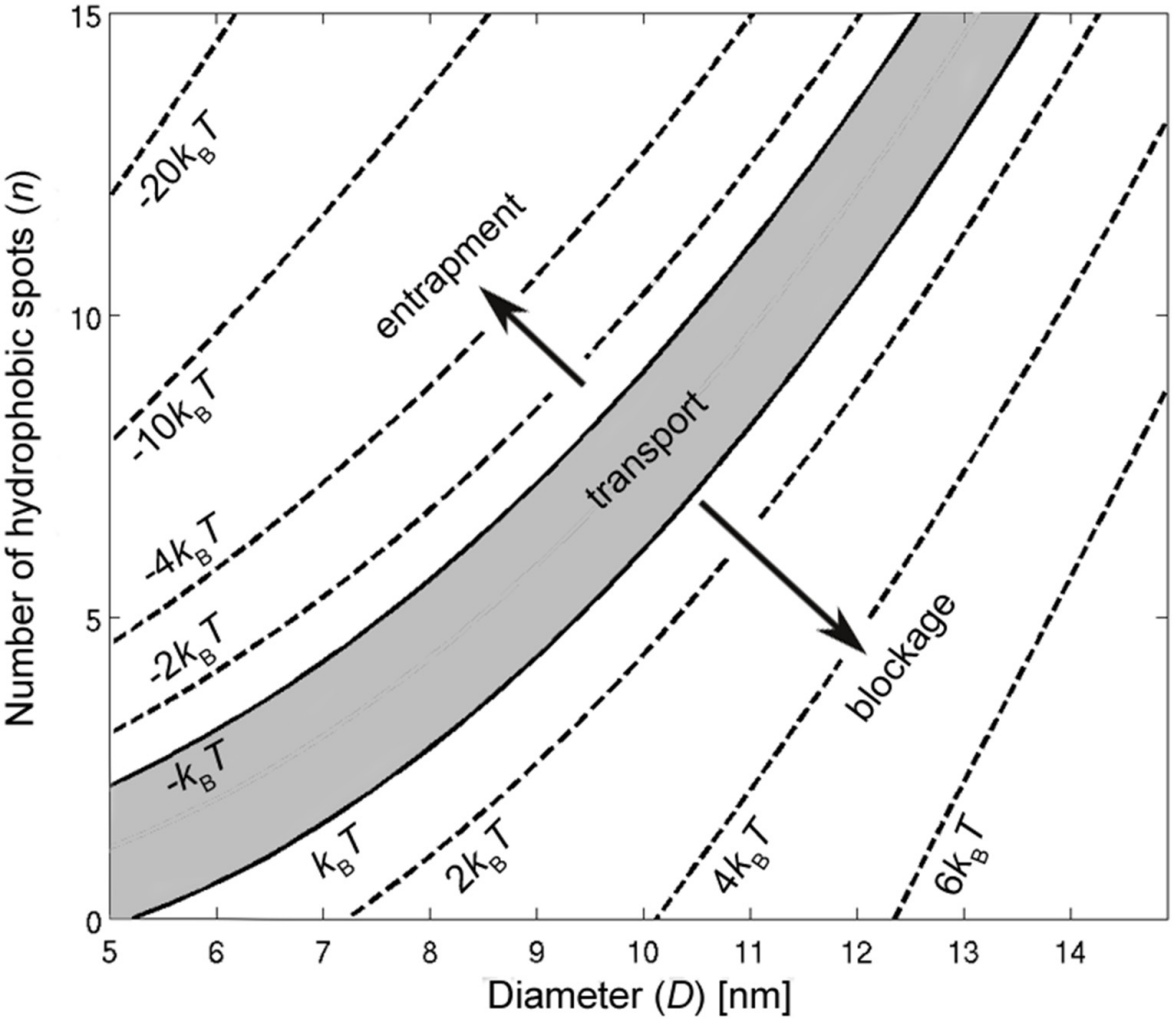
Contour plot of the energy barrier *G* of the NPC as a function of cargo diameter *D* and number of hydrophobic binding spots *n*.

Using the proposed model, the energy barrier of the NPC is characterized for Kap-cargo complexes with different sizes and number of hydrophobic binding spots in Fig 8. The calculated energy map suggests that efficient transport occurs in a strip confined between two iso-lines of +*k*_*B*_*T* and −*k*_*B*_*T* which is shown as a gray area in Fig 8. The region below the +*k*_*B*_*T* line corresponds to the situation in which the number of hydrophobic bindings spots is too small to reduce the free energy barrier enough for transport to be possible. The area above the −*k*_*B*_*T* line represents a state in which the NPC turns into an energy well due to the presence of a large number of hydrophobic binding spots on the cargo. These spots have a high affinity to the FG-nups which results into entrapment of the cargo inside the pore.

## Conclusion

We have characterized the selective permeability barrier of the NPC by studying the energetics of transport through the pore. All 128 FG-Nups of the yeast NPC are accounted for in a one-bead-per-amino acid coarse-grained model in which the FG-Nup amino acid sequence is explicitly represented [37]. The energy barrier for transport of model cargoes has been studied by calculating PMF curves through umbrella sampling. Our results indicate that the disordered FG-Nups inside the NPC form an energy barrier along the central axis of the NPC that does not allow the passage of inert cargo molecules larger than *D* ∼ 5 nm in diameter. However, the PMF curves of Kap-cargo complexes show that the attachment of several hydrophobic binding spots to the surface of the cargo complex lowers the energy barrier below *k*_*B*_*T*, facilitating the transport of large cargo molecules. In addition, the effect of surface hydrophobicity and spacing between binding spots on active transport has been analyzed. Our results show that in addition to the number of hydrophobic spots the spacing between binding spots is a key feature in facilitated transport through the NPC. Additionally, we show that there is an optimal number of hydrophobic binding spots for efficient transport of Kap-cargo complexes of a certain size. Depending on the number and spacing of binding spots, a cargo can be expelled from, transported through or trapped inside the pore.

## Supporting Information

S1 Text The umbrella sampling procedure. (PDF)

S2 Text Parametrization of the transport model. (PDF)

S1 Fig PMF curves along the central axis of the minimal viable NPC for *D* = 10 nm obtained for a spacing of *dz* = 2.0 nm and 1.5 nm between umbrella windows and with 1X and 2X sampling. (EPS)

S2 Fig The convergence of the PMF curve with increasing sampling (1X, 2X and 3X) for the wildtype NPC with *D* = 10 nm. The spacing between the umbrella windows is set to *dz* = 1.5 nm. (EPS)

S3 Fig The predicted and calculated energy barrier *G* for Kap-cargo complexes of *D* = 10 nm and *D* = 7.3 nm with different number of hydrophobic binding spots *n*. (EPS)
