# Supplementary material for: Energetics of Transport through the Nuclear Pore Complex
Source: PLoS One. 2016 Feb 19;11(2):e0148876. doi: 10.1371/journal.pone.0148876 (PMC4764519; doi:10.1371/journal.pone.0148876)
Supplement: S1 Text — (PDF) [file pone.0148876.s001.pdf]

The umbrella sampling procedure

The fundamentals of the umbrella sampling method can be found in [48, 54]. We have used the standard g_wham code from [55] to obtain the free energy profiles from a set of separated umbrella simulations. The initial configurations for the umbrella simulations are extracted from fast pulling simulations in which the cargo is pulled from $z = +27$ to $z = -27$ nm along the central axis of the NPC with a constant velocity $v = 0.006$ nm/ps while it is not allowed to move laterally. The initial configurations for the umbrella simulations are then extracted every dz nm along the z-direction. For each umbrella simulation, the center of mass of the cargo is fixed harmonically with a spring constant of $K = 5.0$ kJ mol^{-1} nm^{-2} and each window is simulated for at least 1.5×10^6 steps. The PMF curves are then derived using a weighted histogram analysis [55, 56]. A parameter study of the spacing between the umbrella windows dz is performed on the minimal viable NPC with a cargo of diameter $D = 10$ nm (see S1 Fig). The minimal viable NPC is the NPC which can perform its function with a minimal amount of FG-nups, see section Size selectivity of the NPC.

The results show that decreasing the spacing between umbrella windows from $dz = 2.0$ to $dz = 1.5$, considerably improves the quality of the PMF curve. The quality can be further improved by sampling each umbrella window several times with a different initial configuration (e.g., 1X means sampling once and 2X means sampling twice). This is done by generating new initial configurations for each umbrella window through separate pulling simulations and simulating each window for at least 1.5×10^6 steps. The data from separate simulations on the same window are combined together to calculate the converged PMF curve. The convergence of the PMF curves for the minimal viable and wildtype NPCs are shown in S1 Fig and S2 Fig, respectively. These PMF curves show a peak close to the center of the NPC (see S1 Fig and S2 Fig). Therefore, the PMF curves for all other cases have been calculated for z-values ranging from -5 to 27 nm to save computer time. In order to obtain PMF curves in the radial direction, the same procedure is followed except that the spacing between umbrella windows is chosen to be 1.2 nm for faster convergence.

References

48. Torrie GM, Valleau JP. Nonphysical sampling distributions in Monte Carlo free-energy estimation: Umbrella sampling. Journal of Computational Physics. 1977;23(2):187–199.

54. Roux B. The calculation of the potential of mean force using computer simulations. Computer Physics Communications. 1995;91(1):275–282.

55. Hub JS, de Groot BL, van der Spoel D. g_wham-A Free Weighted Histogram Analysis Implementation Including Robust Error and Autocorrelation Estimates. Journal of Chemical Theory and Computation. 2010;6(12):3713–3720.

56. Kumar S, Rosenberg JM, Bouzida D, Swendsen RH, Kollman PA. The weighted histogram analysis method for free-energy calculations on biomolecules. I. The method. Journal of Computational Chemistry. 1992;13(8):1011–1021.
