# Supplementary material for: Energetics of Transport through the Nuclear Pore Complex
Source: PLoS One. 2016 Feb 19;11(2):e0148876. doi: 10.1371/journal.pone.0148876 (PMC4764519; doi:10.1371/journal.pone.0148876)
Supplement: S2 Text — (PDF) [file pone.0148876.s002.pdf]

Parametrization of the transport model:

The first term in Eq. (1) is a quadratic function $f(D') = 356.4D'^2$ which is obtained by fitting the data points in Fig. 3. The second term is a quadratic function $g(X) = 150X^2 + 44X + 0.22$ which is obtained by fitting data points of Fig. 7 (see inset) corresponding to a cargo with $D = 10$ nm.

S3 Fig shows a comparison between the transport model of Eq. (1) (solid lines) and the data points from the umbrella sampling simulations (filled circles). The blue data points corresponding to $D = 10$ nm have been used for fitting. The black data points correspond to $D = 7.3$ nm, showing excellent agreement with the predictions (green solid lines) of the transport model.
